# The Unexpected Ovarian Pregnancy at Laparoscopy: A Review of Management

**DOI:** 10.1155/2017/9856802

**Published:** 2017-09-11

**Authors:** Meher Tabassum, Kiran Atmuri

**Affiliations:** Department of Obstetrics and Gynaecology, The Royal Women's Hospital, 20 Flemington Road, Parkville, VIC 3052, Australia

## Abstract

Ovarian ectopic pregnancies are a rare occurrence; however the incidence is on the rise. Preoperative diagnosis remains difficult due to nonspecific clinical symptoms and USS findings. Most patients undergo diagnostic laparoscopy with subsequent surgical management. We present the case of a 32-year-old female who presented with vaginal bleeding and an unsited pregnancy, with a BhCG of 24693. Formal USS described unruptured right tubal ectopic with ovarian pregnancy being diagnosed at laparoscopy. A wedge resection was conducted to preserve ovarian function. Postoperative recovery was uneventful and BhCG levels returned to zero (nonpregnant) in an outpatient setting. Although laparoscopy remains the gold standard of diagnosis and treatment, in this case report we discuss benefits of early diagnosis for fertility conserving management, including nonsurgical options.

## 1. Case Report

**Figure 1:**
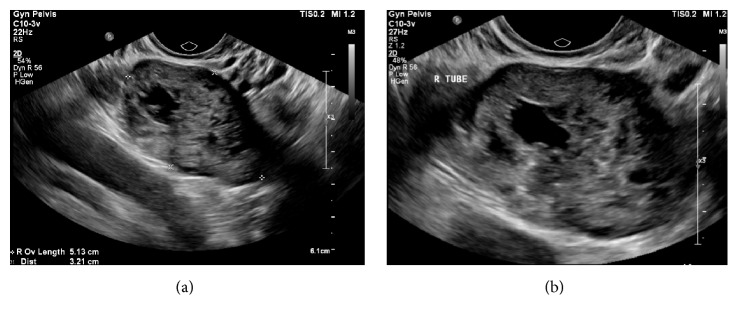
Ultrasound images showing normal right ovary and right tubal ectopic.

A 32-year-old female, gravida 6 and parity 3, presented with mild vaginal bleeding and unsited pregnancy. Her obstetric history included one normal vaginal delivery, followed by 2 caesarean sections and previous left salpingectomy for removal of ectopic pregnancy. On presentation she had been amenorrhoeic for 36 days and was estimated to be at 5 weeks and 6 days of gestation by the first day of her last menstrual period. An outpatient USS requested by her general practitioner 4 days prior to admission demonstrated no intra- or extrauterine pregnancy. On examination she was haemodynamically stable with a soft abdomen. There was mild tenderness in the left iliac fossa but no signs of peritonism. A speculum exam revealed a normal cervix with no evidence of bleeding. On a bimanual examination she was tender in the right adnexa but no masses were palpable. Her serum BhCG was 24693 and Hb 139. A bedside USS was suspicious for a right sided ectopic pregnancy and this was confirmed on a formal USS which described an unruptured right tube ectopic 51 × 36 × 32 mm and a small amount of free fluid in the pouch of Douglas (Figure 1). Both ovaries were reported to appear normal.

**Figure 2:**
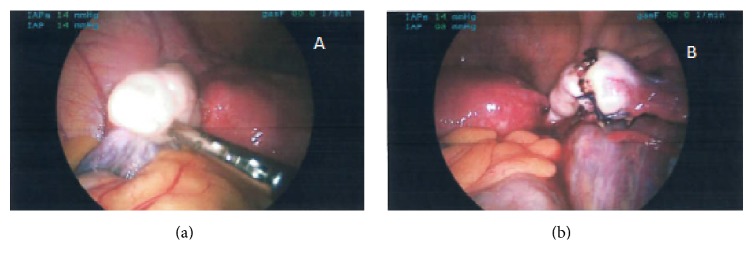
Intraoperative findings. (a) Ovarian ectopic. (b) Wedge resection.

On laparoscopy a right ovarian ectopic was identified (Figure 2). As at least 1/3 of the ovary appeared normal; a wedge resection was conducted using bipolar diathermy and scissors. Two interrupted sutures using vicryl 2.0 were used to achieve haemostasis of bleeding from the right ovary. The left ovary, right tube, and ovarian fossa appeared normal. Total blood loss was estimated at 50 ml.

The patient was monitored overnight and had an uneventful postoperative recovery period. She was discharged the following day with weekly outpatient BhCG tracking through the early pregnancy assessment clinic. Diagnosis was confirmed with histopathology.

## 2. Discussion

The earliest reported case of an ovarian pregnancy was described in the 17th century [1] and although it remains one of the rarest forms of ectopic pregnancies the incidence has been rising, currently estimated to be between 0.5 and 3%. Ovarian ectopics occur by fertilisation of an ovum retained in the peritoneal cavity leading to implantation on the ovarian surface [2]. Although the cause of such implantation anomalies remains unclear, current hypotheses include reflux of fertilised oocyte to the ovary, thickening of tunica albuginea, and tubal dysfunction [2, 3]. The rising incidence of ovarian ectopic pregnancies is associated with increased use of artificial reproductive technology (ART) and intrauterine contraceptive devices (IUCD).

Delayed diagnosis of ovarian ectopic pregnancies can be fatal with massive haemorrhage and carry a risk of oophorectomy with subsequent reduced fertility. As demonstrated in the case discussed, preoperative diagnosis of ovarian ectopic can be challenging as symptoms are nonspecific and ultrasound diagnosis is difficult [3]. In the literature case series have revealed preoperative diagnosis achieved in 11–28% of cases [4, 5].

New advances in ultrasound may lead to earlier detection. A case series regarding the ultrasound appearances of ovarian ectopic pregnancy conducted by Comstock et al. [6] showed that although it was uncommon to see yolk sac or embryo, ovarian pregnancies usually appeared on or within the ovary as a cyst with a wide echogenic outside ring. This can be distinguished from a corpus luteum, which may also have a ring-like appearance, as the majority of the corpus luteum rings appear less echogenic than the ovary itself, whereas for ovarian pregnancies it is greater. Therefore finding such a ring on imaging of suspected ectopic pregnancies should alert the clinician and sonographer of the potential for an ovarian pregnancy.

Currently diagnosis is made using the criteria described by Spiegelberg [7] which includes the fact that the ovary is attached to the uterus by the ovarian ligament, the gestational sac is located at the position of the ovary, the fallopian tube is intact with its fimbria and separated from the ovary, and ovarian tissue is present in the specimen histologically. Unfortunately, as Spiegelberg's criteria can only be established at surgery and not by ultrasonography [4], laparoscopy remains the gold standard of diagnosis and treatment.

Diagnosis is commonly made at surgery, which suggests that clinicians must be confident in identifying diagnostic features of an ovarian ectopic at laparoscopy, and also to consider management steps at laparoscopy after diagnosis. Although ipsilateral oophorectomy is definitive in its management, this is becoming less common in favour of fertility preserving surgical management. These include partial ovariectomy (wedge resection), ovarian cystectomy, or blunt dissection of the trophoblastic tissue [8]. Trophoblastic tissue may persist after conservative surgical management and requires follow-up BhCG tracking postoperatively; however in Odejinmi's case series of 12 patients, no case required further treatment [9]. A retrospective analysis conducted in Korea found that there is a high rate of successful subsequent pregnancy and low rate of recurrent ectopic pregnancy after surgical treatment of ovarian ectopics [10].

After preoperative or intraoperative diagnosis of ovarian ectopic pregnancy, if the patient is clinically stable without significant symptoms, clinicians may offer patients conservative or medical management. This may be suitable in those who may carry operative risks as seen in our case where there were significant pelvic adhesion. In those seeking future fertility, nonsurgical management may preserve ovarian tissue. Medical treatment to halt trophoblast development includes administration of methotrexate, prostaglandins, potassium chloride, and hypertonic glucose [11]. The quality of data is limited to case series and there is no proven superior method. In 2003 Mittal et al. successfully treated an ovarian ectopic with laparoscopy-guided methotrexate injection [12]. Annunziata et al. [13] suggest that the criteria for successful methotrexate therapy are a gestation sac <30 mm, absent fetal cardiac activity, and less than 6 weeks of gestation. Juan and colleagues described a case where etoposide (VP-16) was injected into the gestational sac resulting in complete resolution of the ovarian ectopic pregnancy [14] and suggest that this in addition to systemic methotrexate is a potential option for medical treatment.

In patients who are clinically unstable or have significant symptoms or in whom intraoperative diagnosis is not clear, laparoscopy remains the preferred method of treatment. In our case, although the patient did not present with an acute abdomen, was haemodynamically stable, and had a history of two previous caesarean sections, surgical management was the preferable treatment option given the size of her ovarian pregnancy (>30 mm).

## 3. Conclusion

Although it is a rare occurrence, the incidence of ovarian pregnancy is on the rise. Preoperative diagnosis remains difficult; however USG may assist in early detection. In these cases or in haemodynamically stable patients, medical management should be strongly considered, in order to avoid operative complications and preserve fertility of the patient.
